# Improving OSA screening efficiency with subjective questionnaires: integrating STOP-Bang, ESS, and Berlin

**DOI:** 10.3389/fmed.2025.1581904

**Published:** 2025-07-02

**Authors:** Riken Chen, Yuan Zhang, Weilong Ye, Zhaojun Chen, Weifeng Liao, Huizhao Liao, Tingting Sun, Huan Li, Junfen Cheng, Wang Liu, Weimin Yao, Yitian Yang

**Affiliations:** ^1^The Second Affiliated Hospital of Guangdong Medical University, Zhanjiang, China; ^2^State Key Laboratory of Respiratory Disease, National Clinical Research Center for Respiratory Disease, Guangzhou Institute of Respiratory Health, The First Affiliated Hospital of Guangzhou Medical University, Guangzhou, China

**Keywords:** obstructive sleep apnea (OSA), Berlin questionnaires, diagnosis, Epworth Sleepiness Scale (ESS), STOP-Bang questionnaires

## Abstract

**Objective:**

To compare the predictive value of the STOP-Bang questionnaire, the Epworth Sleepiness Scale (ESS), and the Berlin questionnaire, while evaluating the combined application of these tools in a three-step screening strategy for obstructive sleep apnea (OSA).

**Methods:**

From September 1, 2016, to October 31, 2020, at the Sleep Medicine Center of the First Affiliated Hospital of Guangzhou Medical University, 2,208 suspected OSA patients completed the ESS, STOP-Bang, and Berlin questionnaires and underwent polysomnography (PSG). The sensitivity, specificity, positive predictive value (PPV), negative predictive value (NPV), and receiver operating characteristic (ROC) curves were calculated for each questionnaire, and the accuracy and predictive value of combining the STOP-Bang, ESS, and Berlin questionnaires for OSA screening were analyzed.

**Results:**

Among the individual scales, the Berlin questionnaire had the highest area under the curve (AUC), demonstrating the best diagnostic performance and the highest PPV. The ESS had the smallest AUC and the highest specificity, but the lowest sensitivity and NPV. The STOP-Bang had the highest sensitivity and NPV but the lowest specificity and PPV. When the scales were combined, the AUCs of all combinations were less than 0.7, indicating that the diagnostic performance of the combined scales slightly decreased compared to the individual scales. However, among the combined scales, the AUC of the three-scale combination was higher than that of the two-scale combinations. After combining the questionnaires, specificity and PPV increased, but sensitivity and NPV decreased. Among the two-questionnaire combinations, the sensitivity and NPV of the ESS and STOP-Bang combination were higher than those of the ESS and Berlin questionnaire combination, while specificity and PPV were lower. The combination of all three questionnaires resulted in the highest specificity and PPV, but the lowest sensitivity and NPV.

**Conclusion:**

As the number of scales increases, sensitivity and NPV decrease, while specificity and PPV increase. Therefore, we recommend a three-step strategy, combining a STOP-Bang score of 3, an ESS score of 9, and the Berlin questionnaire to improve the specificity and PPV in screening for OSA.

## 1 Introduction

Obstructive sleep apnea (OSA) is a common sleep disorder characterized by the complete or partial obstruction of the airway during sleep, leading to reduced oxygen levels and disrupted sleep (1). OSA is often caused by obesity and upper airway dysfunction, leading to repeated narrowing or collapse of the throat during sleep, which results in intermittent hypoxia and hypercapnia (2). Increasing evidence indicates that OSA is linked to the development and progression of diseases such as coronary artery disease, heart failure, and stroke (3). Additionally, it also increases the risk of Alzheimer’s disease (AD) (4), and diabetes (5). Reports indicate that globally, 936 million adults aged 30 to 69 years have OSA, with 425 million suffering from moderate to severe forms of the condition (6). Insufficient awareness of OSA among the public and healthcare professionals leads to delayed diagnosis, with studies showing that the vast majority (>80%) of moderate to severe OSA cases remain undiagnosed (7). Untreated OSA patients are at increased risk of cardiovascular diseases and impairments in neurocognitive function and mental health (3, 8–10), and OSA significantly reduces patients’ quality of life (11), potentially leading to premature death (12, 13). However, it is concerning that a large number of OSA cases remain undiagnosed (14, 15), and the prevalence of OSA increases with age (16, 17). Therefore, timely and effective diagnosis and treatment of OSA are crucial to mitigating its adverse health effects, improving quality of life, and reducing mortality.

The gold standard for diagnosing OSA is in-laboratory PSG. However, this method requires a sleep laboratory and skilled personnel to conduct overnight monitoring, making it expensive, technically complex, and time-consuming. Therefore, it is not suitable for widespread use, especially in hospitals in small to medium-sized cities where many patients require testing for suspected OSA (2). Consequently, several simple and efficient screening tools have been developed to identify the risk of OSA, including the NoSAS score (18, 19), STOP-Bang questionnaire (20), GOAL questionnaire (21), Berlin questionnaire (22), and ESS score (23). Previous studies have shown that combining the STOP-Bang questionnaire with the ESS score (24)or the NoSAS score with the ESS score (25) can improve the specificity of OSA diagnosis. However, to date, no studies have investigated the diagnostic performance of combining three commonly used sleep-related questionnaires. Given that the STOP-Bang and Berlin questionnaires emphasize objective clinical features (e.g., snoring, BMI, hypertension), while the ESS reflects subjective daytime sleepiness, we hypothesized that integrating these complementary tools may enhance overall screening efficiency and reduce missed diagnoses. Therefore, the primary objective of this study was to evaluate the predictive performance of combining the STOP-Bang, Berlin, and ESS questionnaires for OSA screening, and to determine whether a stepwise screening approach could provide better diagnostic value than individual questionnaires alone. Given that individual questionnaires often prioritize either objective clinical traits (e.g., STOP-Bang, Berlin) or subjective symptoms (e.g., ESS), this study aims to evaluate whether integrating these complementary tools into a stepwise strategy enhances OSA screening efficiency in high-risk populations, particularly under resource-limited conditions.

## 2 Materials and methods

### 2.1 Selection of study subjects

The study included 2,208 subjects from the Sleep Medicine Center at the First Affiliated Hospital of Guangzhou Medical University, with research conducted from September 1, 2016, to October 31, 2020. The study received ethical approval from the Ethics Committee of the First Affiliated Hospital of Guangzhou Medical University on December 5, 2017 (Ethics Number: 201705), and all subjects signed informed consent forms. Inclusion criteria (must meet all four of the following): (1) Age 18 years or older. (2) Total sleep time greater than 4 h. (3) Ability to act independently and have awareness. (4) Ability to complete the questionnaire. Exclusion criteria (any one of the following results in exclusion): (1) History of psychiatric or psychological disorders. (2) Epilepsy or brain tumors. (3) Long-term or current use of benzodiazepines, barbiturates, or other sedatives and hypnotics. (4) Severe organ failure preventing completion of the test. (5) Previous diagnosis of obstructive sleep apnea hypoventilation syndrome (OSA). (6) Failure to complete the questionnaire. (7) Total sleep time less than 4 h. (8) Patients with predominantly central or mixed apneas were excluded. (9) Daytime sleepiness primarily attributable to external factors such as shift work, fatigue, or irregular sleep schedules.

### 2.2 Study content and procedures

In this study, we collected basic information from 2,208 subjects, including: anthropometric data, demographic data, body measurement data, medical history, personal history, and sleep-related breathing activity. We first assessed the subjects using various scales, and then conducted PSG for further diagnostic clarification. All questionnaires were administered prior to polysomnography (PSG) through face-to-face interviews conducted by trained research staff. Patients were actively guided in completing the questionnaires to ensure accuracy and consistency. This approach helped minimize potential misunderstandings and ensured uniform interpretation of each item across participants. Only participants with a STOP-Bang score ≥3 were subsequently administered the Berlin questionnaire and the Epworth Sleepiness Scale (ESS), in line with our stepwise screening strategy. This design aimed to enhance efficiency and reduce unnecessary burden on low-risk individuals, but may limit the generalizability of Berlin and ESS performance to high-risk populations. Based on the PSG results, subjects were categorized into four groups: AHI <5 events/h (normal group); 5 ≤ AHI < 15 events/h (mild OSA group); 15 ≤ AHI < 30 events/h (moderate OSA group); AHI ≥30 events/h (severe OSA group).

### 2.3 Questionnaires

This study employed five validated screening questionnaires: the Epworth Sleepiness Scale (ESS), Berlin questionnaire, STOP, STOP-Bang, and NoSAS. The Epworth Sleepiness Scale (ESS) consists of 8 items assessing the subject’s likelihood of dozing off in common daily situations. Each item is scored from 0 to 3 (0 = would never doze, 1 = slight chance of dozing, 2 = moderate chance, 3 = high chance), yielding a total score ranging from 0 to 24. A score of 9 or higher indicates a potential risk of obstructive sleep apnea (OSA) (23, 26). The Berlin questionnaire comprises 11 items grouped into three categories: (1) snoring severity and witnessed apneas; (2) daytime sleepiness or fatigue; and (3) history of hypertension or a body mass index (BMI) ≥30 kg/m^2^. A positive score in two or more categories classifies the individual as high risk for OSA. The questionnaire was originally developed for screening OSA in general population settings (27, 28). The STOP questionnaire includes four yes/no items assessing: (1) snoring, (2) tiredness during daytime, (3) observed apnea, and (4) high blood pressure. Each “Yes” response scores 1 point, and a total score of ≥2 suggests a high risk for OSA (29, 30). The STOP-Bang questionnaire expands on the STOP tool by adding four additional demographic and anthropometric factors: B (BMI >35 kg/m^2^), A (age >50 years), N (neck circumference >40 cm), and G (male gender). Each of the eight items is scored as 1 for “Yes” and 0 for “No.” A total score of ≥3 is indicative of high OSA risk (20, 31). The NoSAS score comprises five components: 4 points for neck circumference >40 cm; 3 points for BMI 25–30 kg/m^2^, and 5 points for BMI ≥30 kg/m^2^; 2 points for snoring; 4 points for age >55 years; and 2 points for male gender. The maximum score is 17, and a total of ≥8 points suggests a high likelihood of OSA (32, 33).

### 2.4 PSG

All patients were monitored for at least 7 h using the Alice 5 system (Philips Respironics, United States). On the day of monitoring, patients were instructed to avoid caffeine, alcohol, sedatives, and sleeping pills. The monitoring parameters included EEG—electroencephalogram, EMG—electromyogram, oxygen saturation, EOG—electrooculogram, ECG—electrocardiogram, snoring, oral airflow, nasal airflow, thoracic breathing, and body position. The raw data collected by the machine were then manually analyzed by professional sleep specialists according to the guidelines in the “Manual for the Scoring of Sleep and Associated Events” published by the American Academy of Sleep Medicine (AASM) in 2012 to obtain parameters such as sleep architecture and respiratory events. According to the diagnostic guidelines for OSA, a patient can be diagnosed with OSA if they exhibit primarily obstructive respiratory events and have an AHI of 5 or more events per hour. Based on the AHI, patients were divided into four groups: AHI <5 events/h (normal group); 5 ≤ AHI < 15 events/h (mild OSA group); 15 ≤ AHI < 30 events/h (moderate OSA group); and AHI ≥30 events/h (severe OSA group).

### 2.5 Statistical analysis methods

All data analyses were performed using IBM SPSS Statistics version 29.0.1.0. For continuous data, we used one-way analysis of variance (ANOVA) and post-hoc multiple comparisons for descriptive statistics and between-group comparisons, with results presented as mean ± standard deviation. For categorical data, we used the chi-square test for descriptive statistics and between-group comparisons, with results presented as percentages and counts of categorical variables. To evaluate the diagnostic performance of the five questionnaires and their combinations, we plotted ROC curves and calculated the AUC. Based on the diagnostic results of the five questionnaires and PSG, confusion matrices were constructed, from which sensitivity, specificity, PPV, NPV, and diagnostic odds ratio (DOR) were calculated.

## 3 Results

### 3.1 Baseline data analysis

Descriptive statistics provided an overview of the 2,208 participants (Table 1). Their mean age was 47.68 ± 13.94 years; mean BMI was 26.43 ± 4.08 kg/m^2^; neck circumference (NC) was 38.36 ± 3.92 cm; waist circumference (WC) was 95.13 ± 11.36 cm; AHI was 24.54 ± 26.03 events/h; and minimum oxygen saturation was 78.19 ± 13.48%. The average scores for the five questionnaires (NoSAS, ESS, Berlin, STOP, STOP-Bang) were 8.61 ± 3.86, 7.9 ± 5.76, 1.52 ± 0.90, 1.89 ± 1.07, and 3.52 ± 1.49, respectively. Through intergroup comparisons, we found statistically significant differences among the four groups in terms of age, BMI, NC, WC, AHI, minimum oxygen saturation, and the scores of NoSAS, ESS, Berlin, STOP, and STOP-Bang. However, pairwise comparisons revealed no statistically significant differences in age between normal group and moderate OSA group, normal group and severe OSA group, and mild OSA group and moderate OSA group; no significant difference in BMI between mild OSA group and moderate OSA group; and no significant difference in ESS scores between mild OSA group and moderate OSA group.

**Table 1:** Baseline characteristics.

Variables	ALL	AHI < 5	5 ≤ AHI < 15	15 ≤ AHI<30	AHI ≥ 30	*F*/*x*^2^	*p*
*n*	2,208	677	466	352	713		
Male (*n*, %)	1,722 (78.0)	447 (66.0)	347 (74.5)	281 (79.8)	647 (90.7)	128.071	<0.001
Snore (*n*, %)	2,022 (92.1)	536 (80.2)	440 (94.8)	345 (98.6)	701 (98.3)	192.325	<0.001
Smoking	862 (39.0)	204 (30.1)	176 (37.8)	134 (38.1)	348 (48.8)	51.610	<0.001
Drinking alcohol	563 (25.5)	124 (18.3)	122 (26.2)	100 (28.4)	217 (30.4)	29.214	<0.001
Hypertension	612 (27.7)	130 (19.2)	131 (28.1)	124 (35.2)	227 (31.8)	40.486	<0.001
Diabetes mellitus	172 (7.8)	40 (5.9)	35 (7.5)	36 (10.2)	61 (8.6)	6.880	<0.076
Coronary heart disease	106 (4.8)	28 (4.1)	19 (4.1)	21 (6.0)	38 (5.3)	2.670	<0.445
Cerebrovascular disease	56 (2.5)	16 (2.4)	13 (2.8)	12 (3.4)	15 (2.1)	1.827	<0.609
Rhinitis	538 (24.4)	168 (24.8)	118 (25.3)	87 (24.7)	165 (23.1)	0.909	<0.823
Pharyngitis	318 (14.4)	98 (14.5)	69 (14.8)	57 (16.2)	94 (13.2)	1.839	<0.606
Insomnia	103 (4.7)	44 (6.5)	24 (5.2)	16 (4.5)	19 (2.7)	11.794	<0.008
COPD	49 (2.2)	26 (3.8)	13 (2.8)	4 (1.1)	6 (0.8)	17.038	<0.001
Asthma	95 (4.3)	55 (8.1)	19 (4.1)	12 (3.4)	9 (1.3)	40.759	<0.001
Cough	114 (5.2)	39 (5.8)	30 (6.4)	20 (5.7)	25 (3.5)	6.231	<0.101
Age (years)	47.6 ± 13.944	47.2 ± 14.810	49.6 ± 13.186	49.63 ± 14.088	45.80 ± 13.221	10.207	<0.001
BMI (kg/m^2^)	26.43 ± 4.08	24.75 ± 3.97	26.00 ± 3.50	26.55 ± 3.70	28.25 ± 3.96	97.906	<0.001
NC (cm)	38.36 ± 3.92	36.42 ± 3.85	37.91 ± 3.57	38.62 ± 3.31	40.38 ± 3.47	144.746	<0.001
WC (cm)	95.13 ± 11.36	89.71 ± 10.97	93.84 ± 9.99	95.88 ± 10.20	100.76 ± 10.40	132.434	<0.001
AHI (events/h)	24.54 ± 26.03	1.87 ± 1.45	9.41 ± 2.75	21.79 ± 12.10	57.33 ± 17.37	3315.673	<0.001
LSpO_2_ (%)	78.19 ± 13.48	88.07 ± 6.26	82.77 ± 7.02	78.03 ± 8.64	65.88 ± 14.10	621.733	<0.001
ESS	7.90 ± 5.76	6.21 ± 5.10	7.16 ± 5.16	7.30 ± 5.22	10.28 ± 6.20	69.269	<0.001
STOP	1.89 ± 1.07	1.44 ± 0.89	1.83 ± 0.93	2.05 ± 1.21	2.27 ± 1.06	80.537	<0.001
STOP-Bang	3.52 ± 1.49	2.72 ± 1.32	3.40 ± 1.26	3.78 ± 1.49	4.23 ± 1.37	151.322	<0.001
Berlin	1.52 ± 0.90	0.97 ± 0.84	1.49 ± 0.82	1.69 ± 0.75	1.98 ± 0.78	192.439	<0.001
NoSAS	8.61 ± 3.85	6.52 ± 3.81	8.32 ± 3.45	9.22 ± 3.33	10.47 ± 3.33	151.806	<0.001

Continuous data are presented as mean ± standard deviation, while categorical data are expressed as percentages (%) and the number of cases (*n*). AHI, apnea-hypopnea index; BMI, body mass index; NC, neck circumference; WC, waist circumference; LSpO_2_, lowest oxygen saturation.

### 3.2 Diagnostic performance analysis of individual scales

**Figure 1:**
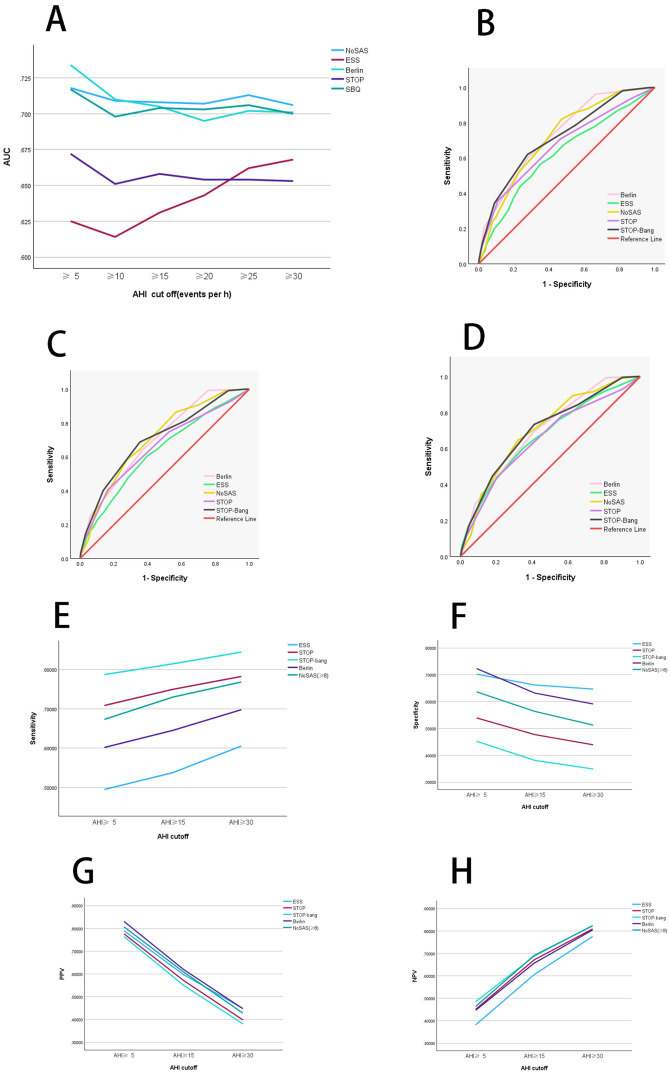
Diagnostic performance analysis of individual scales. **(A)** Comparison of diagnostic performance among independent scales. When AHI was greater than or equal to thresholds of 5 and 10, Berlin shows the highest AUC values. Conversely, AHI was greater than or equal to thresholds of 15, 20, 25, and 30, NoSAS demonstrates the highest AUC values. Thus, Berlin exhibits the best diagnostic performance when AHI was greater than or equal to thresholds of 5 and 10, whereas NoSAS performs best AHI was greater than or equal to thresholds of 15, 20, 25, and 30. **(B)** ROC curves of independent scales at an AHI threshold of 5. When AHI was greater than the threshold of 5, Berlin exhibits superior diagnostic performance compared to other scales. **(C)** ROC curves of independent scales at an AHI threshold of 15. When AHI was greater than the threshold of 15, NoSAS exhibits superior diagnostic performance compared to other scales. **(D)** ROC curves of independent scales at an AHI threshold of 30. When AHI was greater than the threshold of 30, NoSAS exhibits superior diagnostic performance compared to other scales. **(E)** Comparison of sensitivity of independent scales. When AHI was greater than or equal to thresholds of 5, 15, and 30, the sensitivity in descending order is STOP-Bang, Berlin, and ESS. Additionally, as the AHI increases, the sensitivity of the scales also increases. **(F)** Comparison of specificity of independent scales. When AHI was greater than or equal to thresholds of 5, 15, and 30, the specificity in descending order is ESS, Berlin, and STOP-Bang. Additionally, as the AHI increases, the specificity of the scales decreases. **(G)** Comparison of PPV of independent scales. When AHI was greater than or equal to thresholds of 5, 15, and 30, the PPV in descending order is Berlin, ESS, and STOP-Bang. Additionally, as the AHI increases, the PPV of the scales decreases. **(H)** Comparison of NPV of independent scales. When AHI was greater than or equal to thresholds of 5, 15, and 30, the NPV in descending order is STOP-Bang, Berlin, and ESS. Additionally, as the AHI increases, the NPV of the scales increases. AHI, apnea-hypopnea index; PPV, positive predictive value; NPV, negative predictive value; SBQ, STOP-Bang questionnaire; ESS, Epworth Sleepiness Scale; ROC, receiver operating characteristic curve; AUC, area under the curve.

The AUC was determined by plotting the ROC curves (Table 2 and Figures 1A–D). When the apnea-hypopnea index (AHI) is ≥5, ≥15, and ≥30 respectively, the area under the curve (AUC) values of the NoSAS questionnaire are 0.718, 0.708, and 0.706; the AUC values of the Epworth Sleepiness Scale (ESS) are 0.625, 0.631, and 0.668 respectively; the AUC values of the Berlin questionnaire are 0.734, 0.705, and 0.701 respectively; the AUC values of the STOP questionnaire are 0.672, 0.658, and 0.653 respectively; and the AUC values of the STOP-Bang questionnaire are 0.717, 0.704, and 0.700, respectively. Among them, the AUC values of the NoSAS, Berlin, and STOP-Bang questionnaires are all greater than 0.7; the cut-off values of the ESS and STOP questionnaires are all greater than 0.6 and less than 0.7. The larger the cut-off value of the AHI, the smaller the AUC values of the NoSAS, Berlin, STOP, and STOP-Bang questionnaires. The AUC value of the ESS increases as the cut-off value of the AHI increases. When the AHI is ≥5, the AUC value of the Berlin questionnaire is the largest, which is 0.734; when the cut-off values of the AHI are ≥15 and 30, the AUC values of the NoSAS questionnaire are the largest, which are 0.708 and 0.706, respectively. Thus, the Berlin questionnaire demonstrated the best diagnostic performance when AHI was greater than or equal to the threshold of 5, while NoSAS performed best when AHI was greater than or equal to thresholds of 15 and 30.

**Table 2:** Independent scale AUC (95% confidence interval).

AHI cut off	NoSAS	ESS	Berlin	STOP	STOP-Bang
≥5	0.718 (0.694–0.742)	0.625 (0.600–0.649)	0.734 (0.711–0.757)	0.672 (0.648–0.695)	0.717 (0.694–0.740)
≥15	0.708 (0.686–0.729)	0.631 (0.608–0.654)	0.705 (0.684–0.726)	0.658 (0.635–0.681)	0.704 (0.683–0.726)
≥30	0.706 (0.683–0.729)	0.668 (0.643–0.692)	0.701 (0.679–0.724)	0.653 (0.628–0.678)	0.700 (0.677–0.723)

AUC, area under the curve; CI, confidence interval; AHI, apnea-hypopnea index; ESS, Epworth Sleepiness Scale.

After organizing the confusion matrix and calculating, the sensitivity, specificity, PPV, and NPV are obtained (Table 3). When AHI was greater than or equal to thresholds of 5, 15, and 30, the sensitivity of ESS is 0.495, 0.538, and 0.605, respectively; for Berlin, the sensitivity is 0.602, 0.645, and 0.698; for STOP-Bang, the sensitivity is 0.787, 0.814, and 0.844. Sensitivity decreases in the following order: STOP-Bang, Berlin, ESS. Additionally, sensitivity increases with higher AHI values (Figure 1E).

**Table 3:** Predictive parameters of independent scales.

Scale	Sensitivity	Specificity	PPV	NPV	DOR
AHI ≥5					
ESS (≥9)	0.495 (0.470–0.520)	0.702 (0.668–0.737)	0.789 (0.763–0.815)	0.382 (0.355–0.409)	2.312
STOP (≥2)	0.708 (0.686–0.731)	0.539 (0.502–0.577)	0.776 (0.754–0.798)	0.451 (0.417–0.485)	2.839
STOP-Bang (≥3)	0.787 (0.766–0.807)	0.452 (0.415–0.490)	0.764 (0.743–0.785)	0.485 (0.446–0.524)	3.045
Berlin (≥2)	0.602 (0.577–0.626)	0.723 (0.689–0.757)	0.830 (0.808–0.852)	0.446 (0.417–0.476)	3.945
NoSAS (≥8)	0.673 (0.649–0.697)	0.636 (0.600–0.672)	0.806 (0.785–0.828)	0.464 (0.432–0.496)	3.599
AHI ≥15					
ESS (≥9)	0.538 (0.508–0.568)	0.662 (0.634–0.689)	0.597 (0.566–0.629)	0.605 (0.578–0.633)	2.278
STOP (≥2)	0.749 (0.723–0.775)	0.477 (0.448–0.506)	0.572 (0.546–0.598)	0.671 (0.639–0.703)	2.724
STOP-Bang (≥3)	0.814 (0.791–0.837)	0.381 (0.352–0.409)	0.550 (0.526–0.575)	0.687 (0.651–0.723)	2.690
Berlin (≥2)	0.645 (0.616–0.674)	0.632 (0.604–0.660)	0.620 (0.591–0.649)	0.656 (0.628–0.684)	3.117
NoSAS (≥8)	0.730 (0.703–0.756)	0.563 (0.535–0.592)	0.609 (0.582–0.636)	0.691 (0.661–0.721)	3.481
AHI ≥30					
ESS (≥9)	0.605 (0.569–0.641)	0.647 (0.622–0.671)	0.449 (0.417–0.480)	0.775 (0.752–0.799)	2.809
STOP (≥2)	0.782 (0.752–0.812)	0.439 (0.414–0.464)	0.398 (0.372–0.424)	0.809 (0.782–0.836)	2.806
STOP-Bang (≥3)	0.844 (0.817–0.871)	0.349 (0.325–0.373)	0.381 (0.357–0.405)	0.825 (0.795–0.854)	2.894
Berlin (≥2)	0.698 (0.664–0.731)	0.591 (0.566–0.616)	0.448 (0.418–0.477)	0.805 (0.781–0.828)	3.336
NoSAS (≥8)	0.768 (0.737–0.799)	0.512 (0.487–0.538)	0.428 (0.401–0.455)	0.823 (0.798–0.847)	3.477

ESS, Epworth Sleepiness Scale; PPV, positive predictive value; NPV, negative predictive value; DOR, diagnostic odds ratio.

When AHI was greater than or equal to thresholds of 5, 15, and 30, the specificity of ESS is 0.702, 0.662, and 0.647, respectively; Berlins specificity is 0.723, 0.632, and 0.591; STOP-Bangs specificity is 0.452, 0.381, and 0.349. Thus, the specificity is highest for ESS, followed by Berlin, and lowest for STOP-Bang. Additionally, specificity decreases with increasing AHI values (Figure 1F).

When AHI was greater than or equal to thresholds of 5, 15, and 30, the PPV of ESS is 0.789, 0.597, and 0.449, respectively; Berlins PPV is 0.830, 0.620, and 0.448; STOP-Bangs PPV is 0.764, 0.550, and 0.381. Therefore, the PPV is highest for Berlin, followed by ESS, and lowest for STOP-Bang. Additionally, PPV decreases with increasing AHI values (Figure 1G).

When AHI was greater than or equal to thresholds of 5, 15, and 30, the NPV of ESS is 0.382, 0.605, and 0.775, respectively; Berlins NPV is 0.446, 0.656, and 0.805; STOP-Bangs NPV is 0.485, 0.687, and 0.825. Thus, the NPV is highest for STOP-Bang, followed by Berlin, and lowest for ESS. Additionally, NPV increases with higher AHI values (Figure 1H).

Overall, ESS exhibits the lowest sensitivity and NPV but the highest specificity. Berlin shows the highest PPV. STOP-Bang demonstrates the highest sensitivity and NPV, but the lowest specificity and PPV.

### 3.3 Diagnostic performance of combined scales

**Figure 2:**
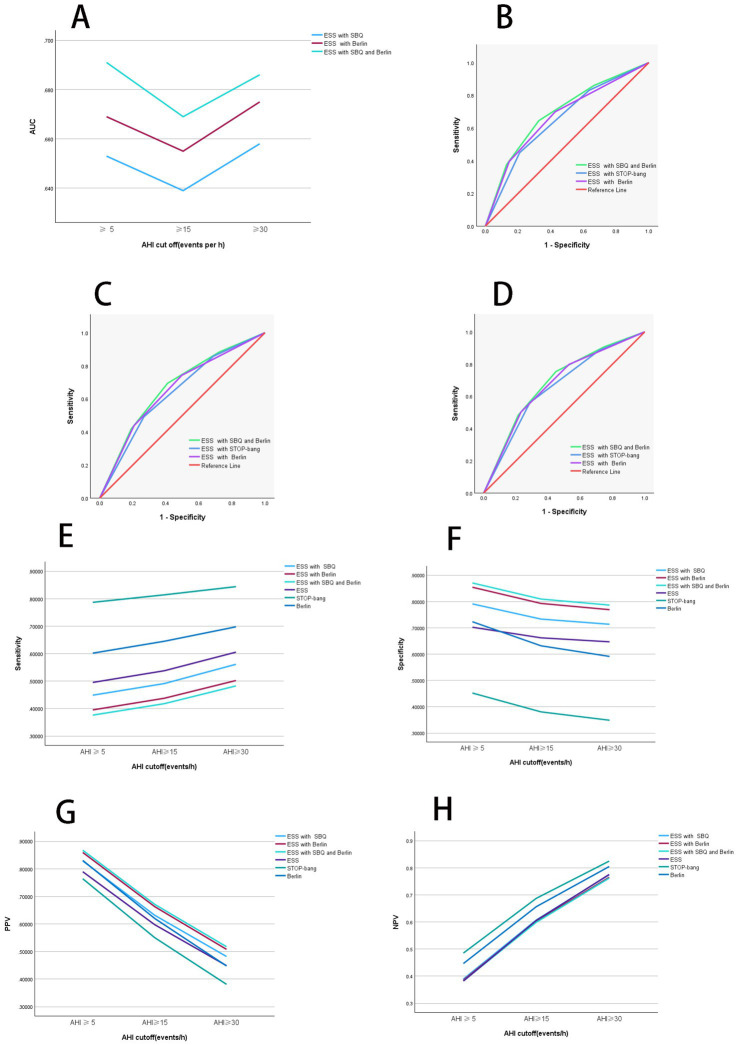
Diagnostic performance of combined scales. **(A)** Diagnostic performance comparison of combined scales. When AHI was greater than or equal to thresholds of 5, 15, and 30, the AUC values rank from highest to lowest as follows: ESS combined with STOP-Bang questionnaire (SBQ), ESS combined with Berlin, and ESS combined with both STOP-Bang and Berlin. At an AHI threshold of 15, the AUC values for all combined scales are lower compared to those at other AHI thresholds. **(B)** ROC curves of combined scales with AHI at 5. When AHI was greater than or equal to the threshold of 5, the combined scales of ESS with Berlin and STOP-Bang show the highest AUC values, indicating the best diagnostic performance. **(C)** ROC curves of combined scales with AHI at 15. When AHI was greater than or equal to the threshold of 15, the combined scales of ESS with Berlin and STOP-Bang exhibit the highest AUC values, indicating the best diagnostic performance. **(D)** ROC curves of combined scales with AHI at 30. When AHI was greater than or equal to the threshold of 30, the combined scales of ESS with Berlin and STOP-Bang exhibit the highest AUC values, indicating the best diagnostic performance. **(E)** Comparison of sensitivity for combined scales. When AHI was greater than or equal to thresholds of 5, 15, and 30, the sensitivity from highest to lowest is ESS, ESS combined with Berlin, and ESS combined with Berlin and STOP-Bang. Sensitivity increases with higher AHI values. **(F)** Comparison of specificity for combined scales. When AHI was greater than or equal to thresholds of 5, 15, and 30, the specificity from highest to lowest is ESS combined with STOP-Bang and Berlin, ESS combined with Berlin, and ESS. Specificity decreases as the AHI increases. **(G)** Comparison of positive predictive value for combined scales. When AHI was greater than or equal to thresholds of 5, 15, and 30, the PPV from highest to lowest is ESS combined with Berlin and STOP-Bang, ESS combined with Berlin, and ESS. PPV decreases with increasing AHI values. **(H)** Comparison of negative predictive value for combined scales. When AHI was greater than or equal to thresholds of 5, 15, and 30, the NPV from highest to lowest is ESS, ESS combined with Berlin, and ESS combined with Berlin and STOP-Bang. NPV increases with increasing AHI values. AHI, apnea-hypopnea index; ESS, Epworth Sleepiness Scale; SBQ, STOP-Bang questionnaire; PPV, positive predictive value; NPV, negative predictive value; ROC, receiver operating characteristic curve; AUC, area under the curve. Summary: In the two-scale combinations, ESS combined with STOP-Bang demonstrates higher sensitivity and NPV compared to ESS combined with Berlin, while showing lower specificity and PPV. When combining all three scales, both specificity and PPV improve, but sensitivity and NPV decrease.

The ROC curves were used to determine the AUC values for the combinations of three scales (ESS, STOP-Bang, and Berlin). The results are illustrated in Figures 2A–D, and summarized in Table 4. The AUC values for all combined scales were found to be greater than 0.6 but less than 0.7.

**Table 4:** Combined scales AUC (95% confidence interval).

AHI cut off (events per h)	ESS with STOP-Bang	ESS with Berlin	ESS with STOP-Bang and Berlin
≥5	0.653 (0.629–0.678)	0.669 (0.646–0.693)	0.691 (0.668–0.715)
≥15	0.639 (0.616–0.662)	0.655 (0.632–0.677)	0.669 (0.647–0.692)
≥30	0.658 (0.634–0.683)	0.675 (0.651–0.699)	0.686 (0.662–0.709)

AUC, area under the curve; CI, confidence interval; AHI, apnea-hypopnea index; ESS, Epworth Sleepiness Scale.

When AHI was greater than or equal to thresholds of 5, 15, and 30, the AUC values for the ESS combined with STOP-Bang were 0.653, 0.639, and 0.658, respectively. The AUC values for the ESS combined with Berlin were 0.669, 0.655, and 0.675 for the same thresholds. The AUC values for the combination of ESS, STOP-Bang, and Berlin were 0.691, 0.669, and 0.686, respectively. The AUC values were ranked from smallest to largest as follows: ESS combined with STOP-Bang, ESS combined with Berlin, and the combination of ESS, STOP-Bang, and Berlin. Notably, the AUC values were lowest When AHI was greater than or equal to threshold of 15.

The sensitivity, specificity, PPV, and NPV of the combined scales were calculated from the confusion matrix (Table 5).

**Table 5:** Predictive value of diagnostic performance for combined scales.

Scale	Sensitivity	Specificity	PPV	NPV
AHI ≥5 events/h				
ESS with STOP-Bang	0.449 (0.424–0.474)	0.791 (0.760–0.821)	0.829 (0.803–0.854)	0.389 (0.363–0.415)
ESS with Berlin	0.395 (0.371–0.420)	0.854 (0.828–0.881)	0.859 (0.833–0.885)	0.385 (0.361–0.410)
ESS with STOP-Bang and Berlin	0.376 (0.352–0.400)	0.870 (0.845–0.896)	0.867 (0.841–0.893)	0.383 (0.358–0.407)
AHI ≥15 events/h				
ESS with STOP-Bang	0.491 (0.461–0.521)	0.733 (0.708–0.759)	0.632 (0.599–0.665)	0.607 (0.581–0.633)
ESS with Berlin	0.438 (0.408–0.467)	0.793 (0.769–0.816)	0.663 (0.628–0.698)	0.602 (0.577–0.627)
ESS with STOP-Bang and Berlin	0.418 (0.388–0.447)	0.809 (0.787–0.832)	0.671 (0.635–0.707)	0.599 (0.574–0.623)
AHI ≥30 events/h				
ESS with STOP-Bang	0.561 (0.525–0.598)	0.713 (0.691–0.736)	0.482 (0.448–0.516)	0.774 (0.752–0.796)
ESS with Berlin	0.502 (0.465–0.539)	0.769 (0.748–0.790)	0.508 (0.471–0.545)	0.765 (0.743–0.786)
ESS with STOP-Bang and Berlin	0.482 (0.446–0.519)	0.786 (0.765–0.807)	0.517 (0.479–0.555)	0.762 (0.741–0.783)

AHI, apnea-hypopnea index; ESS, Epworth Sleepiness Scale; PPV, positive predictive value; NPV, negative predictive value.

When AHI was greater than or equal to thresholds of 5, 15, and 30, the sensitivities of the combined scales are as follows: ESS combined with STOP-Bang showed sensitivities of 0.449, 0.491, and 0.561, respectively; ESS combined with Berlin showed sensitivities of 0.395, 0.438, and 0.502, respectively; and ESS combined with both STOP-Bang and Berlin showed sensitivities of 0.376, 0.418, and 0.482, respectively. The sensitivity ranks from highest to lowest are as follows: ESS combined with STOP-Bang, ESS combined with Berlin, and ESS combined with STOP-Bang and Berlin. It is evident that the sensitivity of the independent scales is higher than that of the two-combination scales, and the sensitivity of the two-combination scales is higher than that of the three-combination scale. Additionally, the sensitivity increases as the AHI threshold increases (Figure 2E).

When AHI was greater than or equal to thresholds of 5, 15, and 30, the specificities of the combined scales are as follows: ESS combined with STOP-Bang showed specificities of 0.791, 0.733, and 0.713, respectively; ESS combined with Berlin showed specificities of 0.854, 0.793, and 0.769, respectively; and ESS combined with both STOP-Bang and Berlin showed specificities of 0.870, 0.809, and 0.786, respectively. The specificity ranks from highest to lowest are as follows: ESS combined with both STOP-Bang and Berlin, ESS combined with Berlin, and ESS combined with STOP-Bang. The three-combination scale shows higher specificity than the two-combination scales, and the two-combination scales show higher specificity than the independent scales (Figure 2F).

When AHI was greater than or equal to thresholds of 5, 15, and 30, the positive predictive values (PPVs) for the combination of ESS and STOP-Bang are 0.829, 0.632, and 0.482, respectively; for ESS combined with Berlin, they are 0.859, 0.663, and 0.508; and for the combination of ESS, STOP-Bang, and Berlin, they are 0.867, 0.671, and 0.517. The PPVs, ranked from highest to lowest, are as follows: ESS combined with STOP-Bang and Berlin, ESS combined with Berlin, and ESS combined with STOP-Bang. The PPV for the triple combination is higher than that for the dual combinations, and the PPV for the dual combinations is higher than that for the individual scales. Additionally, the PPV decreases as the AHI increases (Figure 2G).

When AHI was greater than or equal to thresholds of 5, 15, and 30, the negative predictive values (NPVs) for the combination of ESS and STOP-Bang are 0.389, 0.607, and 0.774, respectively; for ESS combined with Berlin, they are 0.385, 0.602, and 0.765; and for the combination of ESS, STOP-Bang, and Berlin, they are 0.383, 0.599, and 0.762. The NPVs, ranked from highest to lowest, are as follows: ESS combined with STOP-Bang, ESS combined with Berlin, and ESS combined with STOP-Bang and Berlin. The NPV for the individual scales is higher than that for the dual combinations, and the NPV for the dual combinations is higher than that for the triple combination. Additionally, the NPV increases as the AHI increases (Figure 2H).

### 3.4 “Three-step strategy” for screening OSA patients

Initially, all 2,208 patients completed the STOP-Bang questionnaire. Among them, 1,575 patients had a score of ≥3. Of these, 76% had an AHI ≥5, 55% had an AHI ≥15, and 38% had an AHI ≥30. Conversely, 633 patients scored <3; among them, 52% had an AHI ≥5, 31% had an AHI ≥15, and 18% had an AHI ≥30.

Subsequently, patients with a STOP-Bang score ≥3 were asked to complete the ESS questionnaire. Out of these, 836 patients had a score ≥9. Among this group, 83% had an AHI ≥5, 63% had an AHI ≥15, and 48% had an AHI ≥30. The remaining 739 patients scored <9; of these, 61% had an AHI ≥5, 39% had an AHI ≥15, and 23% had an AHI ≥30.

**Figure 3:**
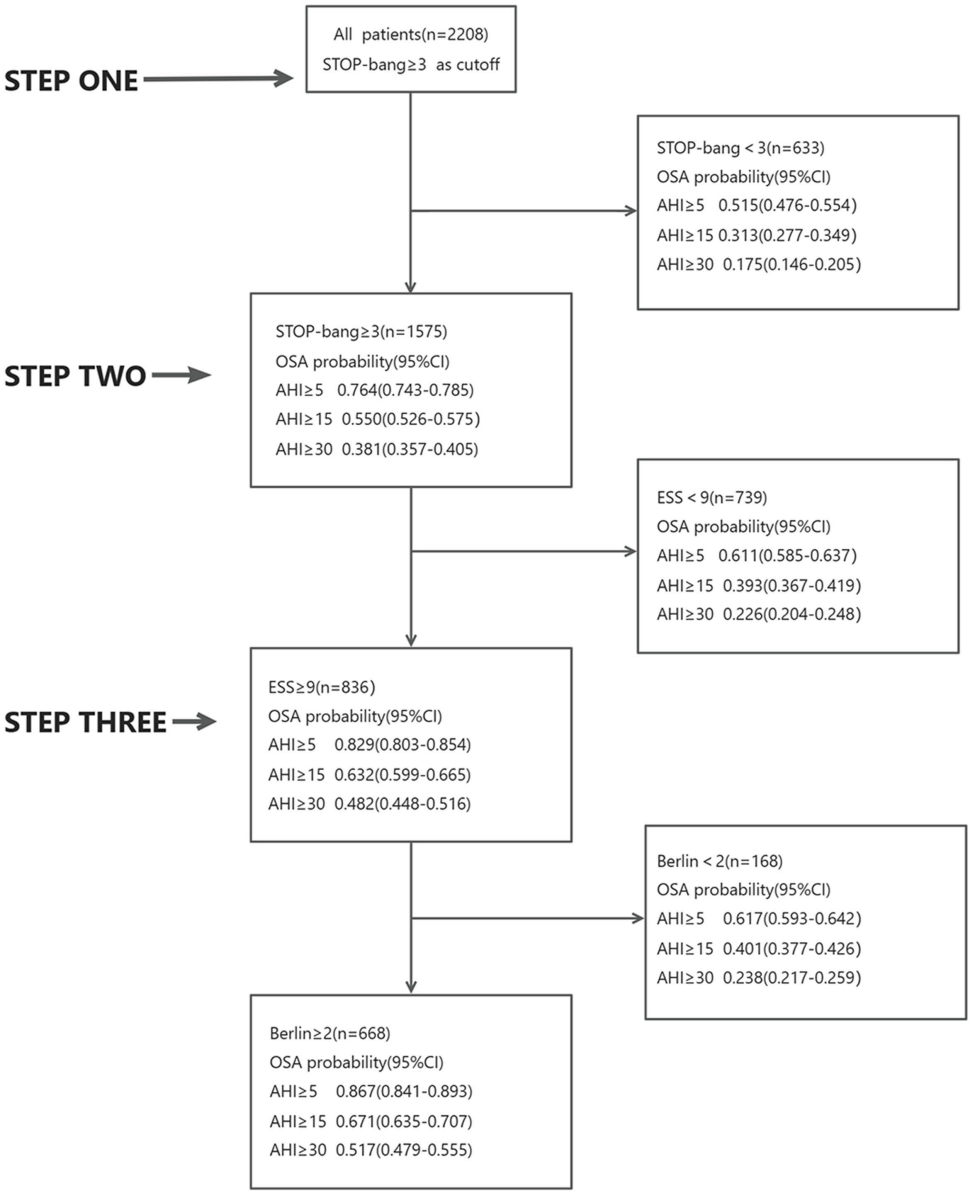
Three-step diagnostic strategy.

Finally, the 836 patients with both STOP-Bang scores ≥3 and ESS scores ≥9 completed the Berlin questionnaire. Among these, 662 patients scored ≥2. In this group, 87% had an AHI ≥5, 67% had an AHI ≥15, and 51% had an AHI ≥30. The remaining 168 patients scored <2; of these, 62% had an AHI ≥5, 40% had an AHI ≥15, and 24% had an AHI ≥30 (Figure 3).

## 4 Discussion

OSA is a condition with a high prevalence and low diagnostic rate. Currently, PSG is considered the gold standard for diagnosing the presence and severity of OSA. However, it is associated with significant time consumption, high costs, and poor patient compliance (34). Therefore, several simple sleep monitoring devices and screening questionnaires have been developed to screen for OSA (35). Factors such as age, gender, body mass index (BMI), neck circumference (NC), waist circumference (WC), hypertension, and coronary artery disease have been shown to be significantly associated with the occurrence of OSA (36). In a meta-analysis, the Berlin questionnaire (BQ), STOP-Bang, STOP, and ESS were found to have relatively low specificity when detecting different severities of OSA (26). Consequently, the search for straightforward screening tools to identify OSA patients has become increasingly important. It has been reported that compared to STOP or ESS, the STOP-Bang questionnaire is more suitable for screening OSA, and it was originally developed for preoperative screening of OSA in surgical patients (26). The Berlin questionnaire was developed by a group of respiratory and primary care physicians to screen high-risk OSA patients (37). An ideal screening tool should have high sensitivity and specificity, as well as a high AUC (38).

In this study, among the 2,208 suspected OSA patients, 1,531 were diagnosed with the condition, with a significantly higher proportion of males compared to females, which aligns with the epidemiological characteristics of OSA. The ROC curve analysis indicated that the AUC values for STOP and ESS were slightly lower than those for NoSAS, Berlin, and STOP-Bang scores. These findings suggest that NoSAS is a simple and effective tool for risk assessment in suspected OSA patients, consistent with previous studies (39–41). By analyzing the confusion matrix, we calculated the sensitivity, specificity, PPV, and NPV. Overall, ESS demonstrated the lowest sensitivity and NPV but the highest specificity; Berlin had the highest PPV; STOP-Bang showed the highest sensitivity and NPV but the lowest specificity and PPV. Our objective was to evaluate the effectiveness of screening suspected OSA patients using single scoring systems, two-combined scoring systems, and three-combined scoring systems. Based on the AUC results and metrics of sensitivity and specificity, we selected the combination of STOP-Bang, ESS, and Berlin scores. In our three-step screening strategy, we initially used STOP-Bang, which had the highest sensitivity among the three scores, and concluded with ESS, which had the highest specificity. This approach was designed to optimize the screening effectiveness for suspected OSA patients.

Interestingly, our analysis showed that the AUC values for each questionnaire remained relatively stable across different AHI thresholds. This may be explained by the fact that the core risk factors assessed—such as snoring, daytime sleepiness, BMI, and hypertension—are prevalent and consistently associated with OSA regardless of severity level. These symptoms may not intensify linearly with increasing AHI, leading to similar discriminative power across severity groups.

Furthermore, we did not perform formal statistical comparisons of AUCs between questionnaires. Since only a single AUC value was derived per ROC curve without repeated measurements or resampling, conventional significance testing (e.g., *t*-tests or ANOVA) was not feasible. Robust statistical comparison of AUCs typically requires bootstrap methods or cross-validation to estimate variability and allow for inference, which was beyond the scope of this study. Future studies could consider such techniques to better compare diagnostic performance across tools.

This study has several advantages. First, our research benefits from a large sample size. Second, all predictive indicators used are common demographic and anthropometric measurements that can be completed in an outpatient setting without requiring additional equipment, invasive procedures, or tests. This significantly reduces the burden on both doctors and patients and helps improve patient adherence to medical advice. Third, the use of these scales allows patients to clearly see the risk factors for their condition, making it easier for them to make lifestyle changes, such as quitting smoking and controlling weight, thus serving as a potential educational tool. Fourth, combining multiple screening questionnaires such as STOP-Bang, ESS, and Berlin can improve the specificity and positive predictive value (PPV) of OSA screening. Each tool has its own strengths and limitations, and using them together allows for a more balanced and accurate assessment. This approach helps reduce missed diagnoses and false positives by compensating for the weaknesses of individual questionnaires. Fifth, all questionnaires employed in this study were validated Chinese versions with demonstrated reliability and diagnostic performance in Chinese populations. Although these instruments were originally developed for western, English-speaking populations, previous studies, including our own work, have confirmed that the translated versions exhibit comparable sensitivity and specificity to those reported in the original validation cohorts (24, 42). This supports their linguistic equivalence and cultural adaptability, and reinforces the applicability of our findings in the Chinese clinical setting. Additionally, although the Epworth Sleepiness Scale (ESS) is not specifically designed as a screening tool for OSA, it remains a widely used instrument to assess excessive daytime sleepiness, which is a key symptom in many sleep disorders. In this study, ESS was used in combination with standard OSA screening questionnaires to provide a more comprehensive assessment of the patient’s sleep-related symptoms. Previous studies have shown that ESS, when used alongside other tools such as STOP-Bang or Berlin, can offer added diagnostic value by capturing subjective symptoms not addressed by the structural components of standard questionnaires (26, 42). Therefore, while ESS alone may lack specificity for OSA, its integration into a multi-dimensional screening approach helps enhance overall predictive accuracy.

However, this study also has certain limitations. Firstly, it is a retrospective study conducted at a single center rather than a multi-center trial. Secondly, patients were originally referred to the sleep medicine center for PSG due to sleep-related breathing disorders, which may have inflated the PPV and affected the evaluation of our predictive parameters. Thirdly, the construction of our nomogram is based solely on demographic and anthropometric data, without considering atypical clinical features, genetic factors, medical history, and other variables. Fourthly, the Berlin and ESS questionnaires were administered only to participants with a STOP-Bang score ≥3. While this stepwise design aligns with clinical logic and helps improve specificity in high-prevalence settings, it may introduce selection bias and limit generalizability to broader populations. Excluding those with STOP-Bang ≤3 could miss mild OSA cases. Future studies should consider applying all questionnaires to the entire cohort to enable unbiased comparison and validate this approach in general populations. Finally, while this study adopted widely recommended cut-off values for each questionnaire based on prior literature, future research should consider exploring optimal cut-off thresholds using ROC-based analyses within specific populations. Such an approach could help refine the sensitivity-specificity balance and improve the screening performance of these tools in clinical practice.

Lastly, although the three-step strategy was intended to improve specificity and prioritization in screening, the added value of the third step—the Berlin questionnaire—may be limited. Our data showed that a proportion of patients excluded at this stage still had moderate-to-severe OSA, indicating a potential risk of underdiagnosis. Future prospective studies are needed to determine whether the marginal benefit of this additional step justifies its use, or if a simplified two-step model might offer comparable effectiveness with greater simplicity.

## 5 Conclusion

The “three-step strategy,” which combines the STOP-Bang score of ≥3, the ESS score of ≥9, and the Berlin questionnaire, significantly improves the specificity and PPV of screening for OSA patients. This approach demonstrates promising potential for clinical implementation.

## Data Availability

The raw data supporting the conclusions of this article will be made available by the authors, without undue reservation.
